# Exploring Variability in Rifampicin Plasma Exposure and Development of Anti-Tuberculosis Drug-Induced Liver Injury among Patients with Pulmonary Tuberculosis from the Pharmacogenetic Perspective

**DOI:** 10.3390/pharmaceutics16030388

**Published:** 2024-03-12

**Authors:** Agnija Kivrane, Viktorija Ulanova, Solveiga Grinberga, Eduards Sevostjanovs, Anda Viksna, Iveta Ozere, Ineta Bogdanova, Maksims Zolovs, Renate Ranka

**Affiliations:** 1 Latvian Biomedical Research and Study Centre, Ratsupites Street 1, k-1, LV1067 Riga, Latviarenate.ranka@rsu.lv (R.R.); 2 Pharmacogenetic and Precision Medicine Laboratory, Pharmaceutical Education and Research Centre, Riga Stradins University, Konsula Street 21, LV1007 Riga, Latvia; 3 Mass Spectrometry Group, Latvian Institute of Organic Synthesis, Aizkraukles Street 21, LV1006 Riga, Latvia; 4 Riga East University Hospital, Centre of Tuberculosis and Lung Diseases, Stopini Region, LV2118 Upeslejas, Latvia; 5 Department of Infectiology, Riga Stradins University, Dzirciema Street 16, LV1007 Riga, Latvia; 6 Statistics Unit, Riga Stradins University, Dzirciema Street 16, LV1007 Riga, Latvia; 7 Institute of Life Sciences and Technology, Daugavpils University, Parades Street 1a, LV5401 Daugavpils, Latvia

**Keywords:** pharmacogenetics, tuberculosis, pharmacokinetics, drug-induced liver injury, next-generation sequencing, liquid chromatography–tandem mass spectrometry

## Abstract

Genetic polymorphisms can exert a considerable impact on drug pharmacokinetics (PK) and the development of adverse drug reactions (ADR). However, the effect of genetic polymorphisms on the anti-tuberculosis (anti-TB) drug, and particularly rifampicin (RIF), exposure or anti-TB drug-induced liver injury (DILI) remains uncertain. Here, we evaluated the relationship between single nucleotide polymorphisms (SNPs) detected in the RIF pharmacogenes (*AADAC*, *SLCO1B1*, *SLCO1B3*, *ABCB1*, and *NR1I2*) and RIF PK parameters, as well as anti-TB treatment-associated DILI. In total, the study enrolled 46 patients with drug-susceptible pulmonary TB. The RIF plasma concentration was measured using the LC-MS/MS method in the blood samples collected pre-dose and 2 and 6 h post-dose, whilst the DILI status was established using the results from blood biochemical analysis performed before and 10–12 days after treatment onset. The genotyping was conducted using a targeted NGS approach. After adjustment for confounders, the patients carrying the rs3732357 GA/AA genotype of the *NR1I2* gene were found to have significantly lower RIF plasma AUC_0–6 h_ in comparison to those with GG genotype, while the difference in RIF plasma C_max_ was insignificant. None of the analyzed SNPs was related to DILI. Hence, we are the first to report *NR1I2* intronic SNP rs3732357 as the genetic component of variability in RIF exposure. Regarding anti-TB treatment-associated DILI, the other preexisting factors promoting this ADR should be considered.

## 1. Introduction

Cost-effectiveness, availability, and high cure rate (around 86%) are the major considerations for the standard 6-month regimen consisting of rifampicin (RIF), isoniazid (INH), pyrazinamide (PZA), and ethambutol (ETB) to remain the first choice for the treatment of drug-susceptible tuberculosis (DS-TB) [1,2]. RIF remains one of the most important drugs in TB treatment; it is able to penetrate granulomatous lesions and acts on both metabolically active and, most importantly, dormant *Mycobacterium tuberculosis* bacteria by selectively inhibiting bacterial DNA-dependent RNA polymerase [3,4,5,6]. The RIF bactericidal activity is concentration-dependent; therefore, it is essential to maintain RIF plasma concentration within the recommended range throughout the treatment course [7]. Many studies investigating anti-TB drug exposure have highlighted a problem of substantial interindividual variability in the RIF pharmacokinetics (PK) at a standard daily dose of 8–12 mg/kg [2,8,9,10,11,12,13,14]. Indeed, the real-world data demonstrated that the RIF plasma concentration tends to be lower than the currently recommended cutoff (8–24 µg/mL) [15], resulting in underexposure rates from 42% to 93% in different settings [9,13,16,17,18]. This RIF PK variability has been attributed to the patient’s biological sex, body weight, nutrition, comorbidities (e.g., HIV infection and *diabetes mellitus*), and drug formulation used [8,9,10,11,12,13,14]. Clinically, underexposure to one or more anti-TB drugs is associated with poor treatment response, relapse, or the development of drug-resistant TB forms requiring a transition to more toxic and less potent second-line drugs and prolongation of treatment [18,19,20,21,22].

Another point for consideration in relation to anti-TB drug exposure is drug-induced liver injury (DILI)—a frequent adverse drug reaction affecting up to 28% of patients with DS-TB receiving the standard four-drug combination regimen recommended by the World Health Organization (WHO) [2,23]. The DILI severity may vary, ranging from an asymptomatic elevation of liver enzymes to acute liver failure and death [23,24]. Among the identified factors predisposing to anti-TB treatment-related hepatotoxicity are older age, female sex, malnutrition, previous history of hepatobiliary disorders, and comorbidities (e.g., viral hepatitis B and C, and HIV infection), as well as anti-TB drug dose at the upper end of therapeutic range and higher exposure [22,23,24,25,26]. Nevertheless, the mechanism of DILI, including RIF-related hepatotoxicity, has a complex and poorly understood nature.

According to the estimates, genetic polymorphisms causing alterations in the function of drug-metabolizing enzymes and transporters may account for 95% of PK variability for certain drugs [27]. As one example, the carriers of the particular set of mutant alleles encoding N-acethyltransferase-2 (NAT2) are proven to have reduced enzymatic activity, which subsequently leads to the excessive accumulation of acetylhydrazine and hydrazine, which are hepatotoxic metabolites, thus contributing to INH-related hepatotoxicity [23,27,28,29]. Association studies of RIF PK and anti-TB treatment-related hepatotoxicity have exposed numerous targets within the RIF metabolic pathways trying to identify clinically relevant single nucleotide polymorphisms (SNPs); those were genes encoding metabolizing enzymes (arylacetamide deacetylase (*AADAC*)), drug transporters (solute carrier organic anion transporter family member 1B1 and 1B3 (*SLCO1B1* and *SLCO1B3*, respectively)), and efflux pumps (ATP binding cassette subfamily B member 1 (*ABCB1*)), and regulatory proteins (nuclear receptor subfamily 1 group I member 2 (*NR1I2*)) [23,30,31,32,33,34,35,36,37,38,39,40,41,42,43,44,45,46,47]. Still, the current evidence regarding the effect of patient genetic background on RIF exposure variability is inconclusive. The observed discrepancy between studies could be explained, at least partly, by considerable differences in the study design and patient characteristics. In addition, studies were conducted in geographically distinct populations, predominantly in the WHO TB-endemic regions, i.e., Sub-Saharan Africa and South-East Asia, whereas information on the other populations is scarce.

Therefore, to fill this gap and elucidate the role of pharmacogenetics in RIF exposure and the potential effect on the development of anti-TB treatment-related hepatotoxicity, this study aimed to investigate (a) the relationship between SNPs detected in the set of the genes (*AADAC*, *SLCO1B1*, *SLCO1B3*, *ABCB1*, and *NR1I2*) and RIF PK parameters; and (b) the relationship between the detected SNPs and the development of anti-TB treatment-associated DILI in Latvian patients with pulmonary tuberculosis (PTB).

## 2. Materials and Methods

### 2.1. Study Design

This retrospective observational study was conducted from April 2017 to May 2023, involving adult patients (18 years and older) diagnosed with PTB who were admitted to the Riga East University Hospital, Centre of Tuberculosis and Lung Diseases (CTLD). All study participants were of European descent.

In order to minimize the impact of other health conditions on the study results, the following exclusion criteria were applied: pregnancy or lactation, history of cancer, other infectious diseases (e.g., HCV, HBV, HIV, and AIDS), and acute or chronic diseases that could alter liver or renal function. All the clinically relevant patient data were obtained from medical records and patient questionnaires and included demographic and anthropometric information; comorbidities and concomitantly used drugs; smoking and alcohol intake habits; and laboratory findings before TB treatment onset and between the 10th and 12th day of the treatment (alanine aminotransferase (ALAT), aspartate aminotransferase (ASAT), total bilirubin, and conjugated bilirubin levels).

The study was approved by the Central Medical Ethics Committee of Latvia (Approval No. 01-29.1/1 and No. 01-29.1.2/1736), the Ethics Committee of the Riga East Clinical University Hospital (Approval No. 24-A/15), and the Scientific Department of the Riga East Clinical University Hospital (Approval No. ZD/08-06/01-21/187). Before enrolment, all patients were familiarized with the study protocol and then asked to provide written informed consent, thus allowing the use of their genetic and phenotypic information for study purposes.

### 2.2. Clinical Sample Collection and Pretreatment Procedure

All patients were subjected to a WHO-recommended treatment regimen for DS-TB [2] and, at the time of clinical sample collection, were receiving ETB (12–25 mg/kg), PZA (20–30 mg/kg), RIF (8–12 mg/kg), and INH (4–6 mg) once a day for 10–12 days. Blood samples for PK analysis were collected pre-dose (0 h) and 2 and 6 h post-dose into vacutainers with EDTA (BD, Plymouth, UK) and immediately centrifuged at 4000 rpm (3488× *g*) for 15 min at 4 °C to separate the plasma. Plasma aliquots were stored at −70 °C until further analysis. During the PK sampling, additional blood aliquots were drawn, and DNA isolation from whole blood samples was performed using the standard phenol–chloroform method [48]. DNA samples were stored at −20 °C until analysis.

### 2.3. Determination of RIF Pharmacokinetic Parameters

A validated liquid chromatography–tandem mass spectrometry (LC-MS/MS) method [49] was employed to determine RIF concentration in the plasma samples. According to the literature [15], the time point 2 h post-dose is appropriate to assess RIF peak plasma concentration (C_max_), while the area under the time–concentration curve from 0 to 6 h (AUC_0–6 h_) was calculated using the linear trapezoidal rule based on data obtained in the three time points: pre-dose (0 h) and 2 and 6 h post-dose.

### 2.4. SNP Detection and Analysis

The targeted next-generation sequencing (NGS) approach was chosen as the most convenient for the large-scale genetic analysis; our previously published protocol [50] was applied with modifications in the amplification step targeting the *AADAC*, *SLCO1B1*, *SLCO1B3*, *ABCB1*, and *NR1I2* genes. Specifically, in total, 40 new primer pairs were constructed using an online-based Primer-BLAST tool (https://www.ncbi.nlm.nih.gov/tools/primer-blast/; accessed on 8 May 2023) to amplify exons and untranslated regions (UTR) with flanking sequences (≥100 bp and ≥500 bp, respectively) (Supplementary Table S1). All other steps, such as library preparation and sequencing, remained unchanged. Sequencing data bioinformatic analysis was completed on the Galaxy online-based platform (https://usegalaxy.org; accessed on 8 May 2023) [51]. The workflow included the following steps: (a) trimming adapter sequences and low-quality reads (sliding window size 4; quality threshold Q < 20; Trimmomatic, v 0.38); (b) mapping reads to the reference sequence GRCh38.p13, GCF 000001405.39 (Map with BWA-MEM, v 0.7.17.1); (c) filtering out unmapped reads and reads shorter than 50 bp (BAM filter, v 0.5.9); (d) removing PCR duplicates (MarkDuplicates, v 2.18.2.2); (e) variant calling in the genome region of interest (quality metrics: variant depth ≥ 10; mapping quality ≥ 10; base quality ≥ 20; fraction of reads supporting the alternative allele to consider position—0.2; number of reads supporting the alternative allele to consider position ≥ 6; FreeBayes, v 1.3.1); and (f) variant filtering (quality metrics: QUAL > 5; VCFfilter, v 1.0.0). All detected SNPs passed visual inspection using the Integrative Genome Viewer web application (https://igv.org/app/; accessed on 8 May 2023) [52] and subsequently were annotated and identified using the wANNOVAR online-based tool (http://wannovar.wglab.org/; accessed on 8 May 2023) [53] and the NCBI dbSNP database (https://www.ncbi.nlm.nih.gov/snp/; accessed on 8 May 2023). Linkage disequilibrium (LD) was calculated for the detected polymorphisms using the Ensembl Linkage Disequilibrium Calculator [54], which utilizes genotyping data from the 1000 Genomes Project database for SNP pairwise comparisons. Polymorphisms were considered in high LD if r^2^ ≥ 0.8. When selecting tagging SNPs, a priority was given to those with minor allele frequency (MAF) > 5% in the NCBI dbSNP database (SNP frequency in the study population 10–90%), located within coding exons, and/or with previous evidence of functional or regulatory consequences reported in the other studies [23,30,31,32,33,34,35,36,37,38,39,40,41,42,43,44,45,46,47].

### 2.5. Assessment of DILI

Liver function was assessed at the baseline and 10–12 days after anti-TB treatment initiation by determining serum levels of ALAT, ASAT, total bilirubin, and conjugated bilirubin. According to the criteria provided by the CTLD clinical laboratory, DILI was defined as mild if ALAT/ASAT levels were 1.5–5 times the upper limit of normal (ULN) (ALAT 60–205 U/L for males and 45–155 U/L for females; ASAT 55–185 U/L for males and 45–155 U/L for females), moderate if ALAT/ASAT levels 5–10 times the ULN (ALAT 205–410 U/L for males and 155–310 U/L for females; ASAT 185–370 U/L for males and 155–310 U/L for females), and severe if ALAT/ASAT levels >10 times ULN (ALAT > 410 U/L for males and > 310 U/L for females; ASAT > 370 U/L for males and > 310 U/L for females). The increased levels of total and conjugated bilirubin (> 19.0 µmol/L and > 3.4 µmol/L, respectively) were only indicative and were not considered in determining DILI severity.

### 2.6. Statistical Data Analysis

The data distribution of quantitative variables was evaluated by the Shapiro–Wilk test and exploring normal Q-Q plots. Accordingly, quantitative variables were presented as the median and interquartile range (IQR), whereas qualitative variables were expressed as a number of observations (percentage). All detected SNPs were assessed for compliance with the Hardy–Weinberg Equilibrium (HWE) by means of the Chi-Square goodness-of-fit test, using genotype frequencies observed in the study. The size of the study population was the reason for collapsing detected genotypes and employing the dominant genetic model (major allele homozygotes versus heterozygotes plus minor allele homozygotes) in the further analyses. The Pearson’s correlation, Mann–Whitney U test, Kruskal–Wallis test, and Fisher’s exact test were used to describe the study population stratified by RIF plasma exposure and DILI status.

The multivariate linear regression was conducted to test the relationship between RIF exposure and the genotyping data after controlling for confounders: biological sex, age, body weight, and RIF dose. Similarly, the logistic regression was used to assess whether the detected genotypes were related to DILI and estimate odds ratios considering biological sex, age, smoking and alcohol consumption status, and ALAT and ASAT levels at the baseline as confounders. The statistical data analysis was performed using the Jamovi software (v 2.3) [55], and the result was considered statistically significant if the *p*-value was less than 0.05.

## 3. Results

### 3.1. Patient Characteristics and RIF Exposure

A detailed characterization of the study population is given in Table 1. Briefly, 46 otherwise healthy patients with PTB were included in the present study; the majority were males (76.1%) and smokers (76.1%), but the median age was 46 years (IQR: 38–55 years). Albeit, the RIF dose received by the patients conformed to the WHO recommendations (median 9.4 mg/kg; IQR: 8.3–10.9 mg/kg) [2]; the PK analysis showed that the median RIF plasma concentration C_max_ at 2 h was 2.09 µg/mL (IQR: 0.39–5.63 µg/mL); and, based on the reference range defined by Alsultan et al. [15], all but four patients (91.3%) were identified as RIF underexposed (C_max_ at 2 h < 8 µg/mL) (Table 1).

**Figure 1:**
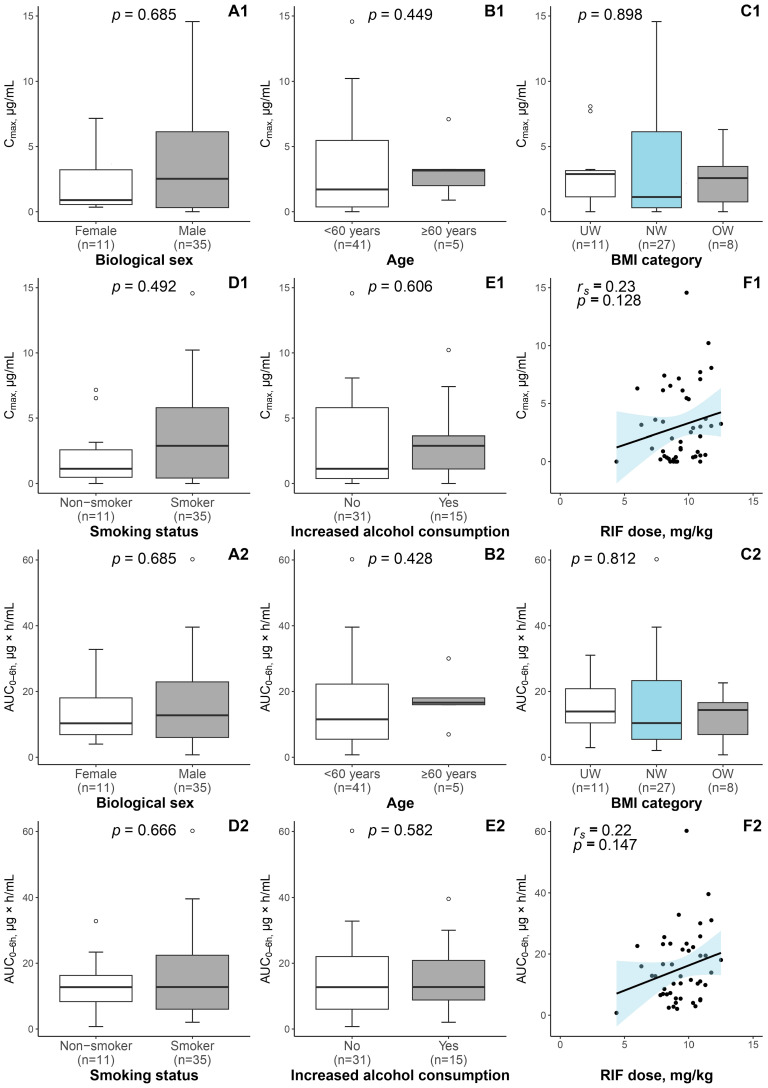
Evaluation of the relationship between RIF PK parameters (1—C_max_; 2—AUC_0–6 h_) and patient characteristics (**A1**–**E1**; **A2**–**E2**). The analysis was performed using the Mann–Whitney U test or Kruskal–Wallis test, where applicable. The margins of the boxes illustrate the first (Q1) and third (Q3) quartiles, whereas the median is indicated with a horizontal line within the box. The top and bottom whiskers represent the highest and lowest values within 1.5 times the IQR. Outliers are plotted as empty dots. The relationship between RIF PK parameters and RIF dose (**F1**; **F2**) was investigated using the Spearman’s correlation; based on a linear model, a trendline with a blue shaded 95% CI was added. The black filled dots are individual measurements. A *p*-value < 0.05 was considered statistically significant. Abbreviations: IQR—interquartile range; BMI—body mass index; RIF—rifampicin; C_max_—peak plasma concentration at 2 h; AUC_0–6 h_—area under the time–concentration curve from 0 to 6 h post-dose; UW—underweight (BMI < 18.5 kg/m^2^); NW—normal weight (18.5 kg/m^2^ < BMI ≤ 25.0 kg/m^2^); OW—overweight (BMI ≥ 25.0 kg/m^2^); *r_s_* = Spearman’s correlation coefficient.

Patient age, biological sex, BMI, smoking status, and self-reported increased alcohol consumption were not significantly associated with the variability in either RIF C_max_ at 2 h or RIF AUC_0–6 h_ (Figure 1). However, among the patient-related factors studied, in comparison to the patients with normal RIF plasma exposure, the underexposed patients had greater body weight (*U* = 142, *p* = 0.021) and thus lower body weight-derived RIF dose (*U* = 26, *p* = 0.021) (Supplementary Table S2).

### 3.2. Results of DILI Assessment

In total, six patients (13.0%) developed mild-to-severe DILI, and in two of them, a slight increase in ALAT/ASAT (up to three times ULN) and conjugated bilirubin levels were observed at the baseline. Clinical symptoms attributable to hepatotoxicity were reported in four patients; three patients had nausea, and two had abdominal pain, while diarrhea, dizziness, and rash were each recorded once. By the end of the study, there were no reports of acute liver failure or fatal cases. When comparing patients in the DILI and non-DILI groups in terms of patient characteristics, RIF dose, and determined PK parameters, significant differences were found solely in the median ALAT and ASAT levels (*U* = 240, *p* < 0.001; *U* = 240, *p* < 0.001) measured in the time frame from 10th to 12th day of anti-TB treatment, whereas the difference in conjugated bilirubin levels was approaching the significance threshold (*U* = 179, *p* = 0.055) (Supplementary Table S3). Regrettably, performing a subgroup analysis after the stratification of patients by the DILI grade was impossible due to a lack of statistical power.

### 3.3. RIF-Associated Pharmacogene SNP Detection

Overall, sequencing data were generated for five RIF-associated pharmacogenes, i.e., *AADAC*, *SLCO1B1*, *SLCO1B3*, *ABCB1*, and *NR1I2*, for 46 patients. Of all polymorphisms detected, 10 SNPs were selected for further analyses based on the criteria outlined in the Materials and Methods section (*SLCO1B1* gene: rs2306283, rs11045819, and rs4149056; *SLCO1B3* gene: rs60140950; *ABCB1* gene: rs9282564 and rs1045642; and *NR1I2* gene: rs3814055, rs3732357, rs2276707, and rs3732359). Table 2 summarizes information on the genomic coordinates, type of nucleotide substitution, and calculated allele frequencies for each SNP. The SNPs were located within coding exons, except for the *NR1I2* gene: a rare exonic SNP was detected in a single patient (rs61755051, C > T; MAF < 1%), so that, for the *NR1I2* gene, intronic and UTR polymorphisms were included in the analysis. In contrast, none of the detected polymorphisms located upstream and downstream of the *AADAC* transcription initiation site, including one exonic SNP carried by 45 out of 46 patients (rs1803155, G > A; MAF > 5%), was included in the analyses primarily due to excessively high (in more than 90% of patients) or low (in less than 10% of patients) variant frequency found in our study population. For all 10 selected SNPs, the observed genotype frequencies conformed to those predicted by the HWE (Table 2).

### 3.4. Relationship between the Detected SNPs and the RIF Pharmacokinetic Parameters

The potential impact of the selected polymorphisms on the RIF PK parameters was investigated under the dominant genetic model, and the obtained results are shown in Table 3. Overall, none of the SNPs located within genes encoding drug transporters (i.e., *SLCO1B1*, *SLCO1B3*, and *ABCB1*) was related to the C_max_ and AUC_0–6 h_ when considering biological sex, age, body weight, and RIF dose as confounders (*p* > 0.05). However, a relationship was found between one of the *NR1I2* gene intronic polymorphisms, i.e., rs3732357, and RIF plasma exposure: the results showed that patients with the GA/AA genotype had lower RIF AUC_0–6 h_ in comparison to the GG genotype (*p* = 0.026). The trend towards significance was also identified between the rs3732357 genotype and RIF concentration in blood plasma 2 h post-dose (*p* = 0.077).

### 3.5. Relationship between the Selected SNPs and the Development of DILI

The genotype frequencies for the selected *SLCO1B1*, *SLCO1B3*, *ABCB1*, and *NR1I2* polymorphisms were compared between the patients with and without DILI, considering the demographic characteristics, self-reported lifestyle factors, and baseline ALAT and ASAT levels as confounders. In general, the regression analysis did not reveal any relationship between the studied SNPs and the development of DILI when assessed under the dominant genetic model (Table 4).

## 4. Discussion

At first, we evaluated RIF exposure in our study population of Latvian patients with PTB. The median RIF plasma concentration 2 h post-dose (C_max_) was 2 µg/mL, which is nearly four times below the lower end of the target range (8–24 µg/mL), using the currently recommended RIF dose [2,15]. An underexposure rate of 91% confirms an alarming tendency of subtherapeutic RIF plasma concentration reported elsewhere and highlights the well-documented but obscure PK variability of anti-TB drugs [9,13,16,17,18]. The underexposed patients had higher a body weight and, consequently, lower body weight-derived RIF dose. However, we did not detect the impact of other frequently discussed determinants of anti-TB drug plasma exposure, as the study population comprised otherwise healthy adult patients, 11% of whom were aged 60 years or older, and the majority were males (76%) [8,9,10,11,12,13,14].

Three out of four drugs in the regimen for treatment of DS-TB, namely RIF, PZA, and INH, induce hepatotoxicity via multiple different mechanisms as early as in the first weeks of the treatment, with half of the cases observed within the first 14 days [57,58]. Additionally, symptoms referable to RIF-related hepatotoxicity may appear sooner than in the case of the other anti-TB drugs [57]. In our study population, 13% of the patients presented with DILI after 10–12 days of anti-TB therapy, which is theoretically consistent with the incidence reported worldwide [23]. Nevertheless, this result should be interpreted carefully, as the definition of DILI may vary between studies. The homogeneity of the study population mentioned earlier was also evident when comparing patient characteristics in the DILI and non-DILI groups, thus raising the question of the underlying molecular mechanisms promoting hepatotoxicity.

In our study, we evaluated 10 SNPs located in the *SLCO1B1*, *SLCO1B3*, *ABCB1*, and *NR1I2* genes as genetic determinants of RIF PK, as well as investigated the possible role of these polymorphisms in the development of anti-TB treatment-related hepatotoxicity since these genes and their encoded products are recognized as being of pharmacogenetic importance. Although the information on *AADAC* genetic polymorphisms is limited, initially, it was considered for analysis because the encoded microsomal enzyme is responsible for the formation of non-toxic 25-desacetyl derivatives of rifamycins, which can be later excreted by bile and eliminated via feces [6,59]. The two groups of investigators have reported on the lower clearance and higher plasma concentration of RIF analogue rifapentine in patients with rs1803155 AA genotype, which may be related to patient race [42,45]. The results of the in vitro study conducted by Shimizu and colleagues [60] suggested that AADAC*3/*3 carriers may have decreased enzymatic activity compared to other diplotypes (AADAC*1/*1 (wild-type—wt), AADAC*1/*2, and AADAC*2/*2). Unfortunately, all detected polymorphisms, including exonic SNP rs1803155, were excluded from the further analysis in our study due to insufficient statistical power resulting from the inadequate variant frequency in the study population. Moreover, the AADAC*3 haplotype consists of two SNPs, the common rs1803155 (MAF > 5%) and the rare rs61733692 (MAF < 1%); so, in a low-endemic setting, there would be a relatively small chance of detecting a sufficient number of patients with AADAC*3/*3 diplotype required to test the hypothesis.

The *SLCO1B1* and *SLCO1B3* transporters are expressed on the sinusoidal membrane of hepatocytes and contribute to the hepatic uptake of RIF from the bloodstream [61]. During in vitro experiments, some *SLCO1B1* genetic polymorphisms have shown promising results, i.e., diminished transporter activity altering RIF exposure [61]. In clinical practice, Allegra et al. [37] reported the *SLCO1B1* rs4149056 TT genotype (wt) to be a predictor of the increased RIF peak plasma concentration. The opposite effect, with lowered plasma concentration, was found in the Ghanaian children bearing two mutant alleles of rs2306283 when performing a subgroup analysis [38]. In the two other studies, rs11045819 was associated with RIF plasma exposure; the other covariates identified were the patient’s geographical origin and biological sex [30,34]. Nevertheless, we did not observe the impact of these three exonic *SLCO1B1* SNPs, as did other authors [31,39,40,43]. In the case of *SLCO1B3*, the exonic polymorphism rs60140950, which was previously described to lower *SLCO1B3* protein expression without any change in transporter function and increase telmisartan plasma concentration, also did not affect the RIF plasma concentration in our patients with PTB [61,62,63].

In the context of DILI, one of the proposed mechanisms of the cholestasis is the competitive inhibition of *SLCO1B1* and *SLCO1B3* transporters by RIF, thereby impairing hepatic uptake of bilirubin—their endogenous substrate [61,64,65]. To our knowledge, there are many studies which have assessed *SLCO1B1* gene polymorphisms as potential contributors in the development of anti-TB treatment-related hepatotoxicity, with conflicting results [32,33,35]. In our work, we failed to replicate the relationship between SNPs comprising the SLCO1B1*15 haplotype (rs2306283 + rs4149056) and RIF-related hepatotoxicity and likewise observe the effect of rs11045819. Interestingly, Zhang et al. [66] reported that serum bilirubin level is influenced by the *SLCO1B1* diplotype, whilst an increase in the serum bilirubin level after low-dose RIF use did not depend on genetic background, suggesting that RIF administration may aggravate pre-existing hyperbilirubinemia and liver impairment but not to be their primary cause. Instead, the *SLCO1B3* polymorphisms have mostly been studied within the frame of taxane toxicity, while the level of evidence supporting particular drug–variant combinations remains low [67,68]. In the present study, the polymorphism rs60140950 was not related to anti-TB treatment-associated DILI.

The *ABCB1* gene encodes the ATP-dependent efflux transporter P-glycoprotein, which mediates the unidirectional transport of xenobiotics, including RIF, from intra- to extracellular space in various tissues and accordingly limits cellular uptake and the distribution of foreign compounds [69]. Regarding the effect of our two investigated *ABCB1* polymorphisms on RIF PK, Huerta-García et al. [39] reported that the rs1045642 AA genotype (wt), along with other patient-dependent factors, accounts for lowered RIF plasma exposure. The functional assays did not provide clear evidence of the effect of this polymorphism on mRNA and protein expression, while the reported association was not re-established in our study or in the other studies [30,31,37,40,43,69]. Similarly, P-glycoprotein harboring the polymorphism rs2032582 from the same haplotype block has shown altered transport activity in vitro, but in the clinical setting, carriers of this polymorphism presented with insignificantly lower RIF plasma clearance [31,69]. The other *ABCB1* polymorphism we investigated, rs9282564, was previously documented to increase tacrolimus plasma concentration [70], but no effect was observed in the case of RIF. When assessing the potential impact of *ABCB1* polymorphisms on the development of DILI in TB-HIV co-infected patients receiving efavirenz and RIF-containing regimens, Yimer and colleagues showed that the rs1045642 GG genotype increased susceptibility to hepatotoxicity independently of concomitant RIF use [32]. On the contrary, in HIV patients without TB co-infection, the rs1045642 mutant allele demonstrated a protective effect, thus proving the complexity of DILI mechanisms, especially in patients with comorbidities [71]. Nevertheless, conflicting results on rs1045642 have been shown in patients with TB and suspected INH-related hepatotoxicity but without other chronic conditions reported [72,73]. Also, our findings do not support the relationship between *ABCB1* polymorphisms, rs1045642 and rs9282564, and anti-TB drug-related hepatotoxicity.

The product of the *NR1I2* gene (also known as *PXR*) belongs to the nuclear receptor family and holds transcriptional regulator functions extending to numerous phases I and II drug-metabolizing enzymes and transporters [74]. In addition, RIF is one of the most potent ligands upregulating the transcription of *NR1I2* downstream genes, and this is thought to partially explain RIF autoinduction phenomena, which is estimated to cause up to 40% of the reduction in RIF plasma concentration in the first weeks of anti-TB treatment [74,75]. In the present study, we assessed the effect of the *NR1I2* intronic and UTR SNPs on RIF PK and found that patients with rs3732357 GA and AA genotypes had significantly lower RIF AUC_0–6 h_ compared to GG genotype (wt) carriers. Using midazolam as a model substrate, He et al. [76] detected changes in CYP3A activity in the presence of this polymorphism, but the impact on RIF plasma exposure had not been described previously. The remaining polymorphisms, which were associated with higher CYP3A activity in African Americans (rs3732359 AA genotype), with higher *NR1I2* promoter activity in vitro (rs3814055 T allele), or substantially increased CYP3A expression and, in turn, altered tacrolimus plasma concentration (rs2276707 T allele), did not exhibit a significant relationship in our study [77,78,79,80].

Calcagno et al. [81] reported that intronic rs2472677 affects INH but not RIF plasma exposure in TB-HIV co-infected patients, while a recently published paper demonstrated its association with a 25% reduction in RIF plasma concentration in patients with TB using a moxifloxacin-containing regimen [46]. This SNP is located in the binding site of hepatic nuclear factor 3β (HNF3β) and is characterized by increased *NR1I2* mRNA levels and CYP3A4 activity; unfortunately, due to limitations of the employed NGS protocol, the effect of rs2472677 was not evaluated in our study [82]. The current evidence indicates the possible involvement of *NR1I2* in the pathogenesis of RIF and INH co-treatment-related hepatotoxicity via RIF-induced upregulation of CYP2E1 and aminolevulinic synthase-1 (ALAS1) expression, leading to excessive production of hepatotoxic INH intermediates, which exacerbates INH-induced oxidative stress, and accumulation of the heme precursor protoporphyrin Ⅸ, responsible for cholestatic liver injury [28,83]. Concerning the impact of *NR1I2* genetic polymorphisms on the development of DILI, the four investigated SNPs (rs3814055, rs3732357, rs2276707, and rs3732359) did not yield any relationship in the present study. Meanwhile, the studies conducted with patients of Asian ancestry reported controversial conclusions. Zazuli et al. [36] and Wang et al. [47] showed that patients carrying the rs3814055 TT genotype or T allele in conjunction with NAT2 non-slow acetylator status, respectively, are more susceptible to anti-TB treatment-associated DILI, but another group of investigators described a protective effect [41]. The protective effect recently reported for rs2276707 under the recessive genetic model has not been replicated yet [44]. Again, two *NR1I2* gene SNPs that were beyond the scope of our study, namely rs7643645 and rs2461823, are believed to affect HNF binding sites and modulate the risk of anti-TB treatment-related hepatotoxicity and severity of non-alcoholic fatty liver disease [41,44,84].

Considering that the anti-TB treatment relies on the administration of drug combinations, implying a wide range of factors affecting drug exposure and occurrence of adverse events, the advantage of our study is the engagement of patients without other severe health conditions, thus limiting any resulting bias, e.g., from comorbidities. Moreover, controlling for relevant cofounders during the statistical data analysis allowed us to reduce variability within the study population. As already mentioned, the pool of studied polymorphisms was limited by the scope of our NGS protocol and sample size. Increasing the study cohort is unlikely to improve the statistical power for those polymorphisms with low frequency in the population of European ancestry in conjunction with low TB incidence within the WHO European Region. Nevertheless, we were able to assess the impact of polymorphisms previously unreported in the present context. Lastly, there is a possibility that an evaluation of blood biochemical parameters in the first weeks of anti-TB treatment bears the risk of missing DILI cases developed lately; however, according to the literature data, signs and symptoms of RIF-related hepatotoxicity usually manifest within this short period.

To summarize all the abovementioned information, we found that the intronic polymorphism rs3732357 in the *NR1I2* gene is related to RIF plasma exposure, whereas none of the studied SNPs in the RIF-associated pharmacogenes, i.e., *SLCO1B1*, *SLCO1B3*, *ABCB1*, and *NR1I2*, was related to the anti-TB drug-induced liver injury in Latvian patients with PTB.

## 5. Conclusions

Our findings, together with previous reports, suggest that the biological effects of the analyzed SNPs are rather insignificant or minor and, thus, do not have a pivotal role in RIF disposition and the mechanism of anti-TB treatment-related hepatotoxicity, which appears to be more likely affected by other patient-dependent factors discussed elsewhere.

Further studies are warranted to include (a) in vitro assays for SNPs lacking functional data to clarify their impact on RIF PK and DILI mechanisms; (b) an analysis of other biomarkers to discriminate liver injury patterns and to speculate on liver injury primarily caused by RIF, as it may cause intrahepatic cholestasis in some patients; and (c) confirmation of the observed relationship between *NR1I2* intronic polymorphism rs3732357 and RIF PK parameters in the other populations.

## Figures and Tables

**Table 1:** Characterization of the study population (n = 46).

		No./Total (%)	Median (IQR)
*Demographic and anthropometric characteristics*			
Biological sex	Male	35/46 (76.1)	
	Female	11/46 (23.9)	
Age, years			46 (38–55)
	<60 years	41/46 (89.1)	
	≥60 years	5/46 (10.9)	
Body weight, kg			64 (55–73)
BMI ^a^	Underweight	11/46 (23.9)	
	Normal weight	27/46 (58.7)	
	Overweight	8/46 (17.4)	
Smoking status	Smoker	35/46 (76.1)	
	Non-smoker	11/46 (23.9)	
Increased alcohol consumption	Yes	15/46 (32.6)	
	No	31/46 (67.4)	
*Baseline blood biochemical parameters*			
ALAT, U/L			17 (13–25)
ASAT, U/L ^b^			18 (15–27)
Total bilirubin, µmol/L ^c^			6.3 (5.2–8.7)
Conjugated bilirubin, µmol/L ^b^			3.2 (2.4–4.0)
*Rifampicin-related data*			
RIF dose, mg/kg			9.4 (8.3–10.9)
RIF C_max_, µg/mL			2.09 (0.39–5.63)
	≥8 µg/mL	4/46 (8.7)	
	<8 µg/mL	42/46 (91.3)	
RIF AUC_0–6 h_, µg × h/mL			12.75 (6.29–22.34)

^a^ According to the World Health Organization recommendations [56], a patient was classified as underweight if the BMI was < 18.5 kg/m^2^ and overweight if the BMI was ≥ 25.0 kg/m^2^. ^b^ Data were available for 44 patients. ^c^ Data were available for 45 patients. Abbreviations: RIF—rifampicin; BMI—body mass index; ALAT—alanine transaminase; ASAT—aspartate aminotransferase.

**Table 2:** Characterization of the single-nucleotide polymorphisms detected in the RIF pharmacogenes (*SLCO1B1*, *SLCO1B3*, *NR1I2*, and *ABCB1*) and observed allele frequencies in the study population (n = 46).

Gene	SNP ID ^a^ (Nucleotide Change)	Genomic Coordinates ^b^	Region ^c^	Type of Substitution	Allele Frequency	HWE *p*-Value ^d^
*SLCO1B1*	rs2306283 (A > G)	chr12:21176804	Exon 5	nonsyn.	A (0.65) G (0.35)	0.713
	rs4149056 (T > C)	chr12:21178615	Exon 6	nonsyn.	T (0.84) C (0.16)	0.810
	rs11045819 (C > A)	chr12:21176879	Exon 5	nonsyn.	C (0.92) A (0.08)	0.577
*SLCO1B3*	rs60140950 (G > C)	chr12:20875274	Exon 9	nonsyn.	G (0.92) C (0.08)	0.577
*ABCB1*	rs1045642 (A > G)	chr7:87509329	Exon 28	syn.	A (0.57) G (0.43)	0.676
	rs9282564 (T > C)	chr7:87600124	Exon 4	nonsyn.	T (0.88) C (0.12)	0.357
*NR1I2*	rs3814055 (C > T)	chr3:119781188	UTR5	N/A	C (0.62) T (0.38)	0.401
	rs2276707 (C > T)	chr3:119815306	Intron 6	N/A	C (0.78) T (0.22)	0.474
	rs3732357 (G > A)	chr3:119812011	Intron 4	N/A	G (0.35) A (0.65)	0.351
	rs3732359 (G > A)	chr3:119817582	UTR3	N/A	G (0.33) A (0.67)	0.457

^a^ SNP ID based on the NCBI dbSNP database (https://www.ncbi.nlm.nih.gov/snp/; accessed on 8 May 2023). ^b^ Reference genome: GRCh38.p13, GCF 000001405.39. ^c^ Following transcripts were used to locate region containing SNP: SLCO1B1: NM_006446.5; SLCO1B3: NM_019844.4; NR1I2: NM_003889.4; ABCB1: NM_001348944.4. ^d^ HWE was tested using the Chi-Square goodness-of-fit test based on the genotype frequency detected in the study. A *p*-value of < 0.05 was considered statistically significant. Abbreviations: RIF—rifampicin; *SLCO1B1*—solute carrier organic anion transporter family member 1B1; *SLCO1B3*—solute carrier organic anion transporter family member 1B3; *ABCB1*—ATP binding cassette subfamily B member 1; *NR1I2*—nuclear receptor subfamily 1 group I member 2; SNP—single nucleotide polymorphism; syn.—synonymous nucleotide substitution; nonsyn.—synonymous nucleotide substitution; N/A—not applicable; UTR—untranslated region; HWE—Hardy–Weinberg Equilibrium.

**Table 3:** Genotype frequencies of the selected SNPs and comparison of the RIF PK parameters between genotype groups.

Gene	SNP ID ^a^	Genotype ^b^	Genotype Frequency, n (%)	Pharmacokinetic Parameters			
				C_max_, µg/mL ^c^	*p*-Value ^d^	AUC_0–6 h_, µg × h/mL ^c^	*p*-Value ^d^
*SLCO1B1*	rs2306283	AA	19 (41.3)	1.13 (0.37–6.13)	0.145	10.39 (5.21–22.25)	0.148
		AG + GG	27 (58.7)	3.06 (0.39–5.47)		15.98 (6.95–22.62)	
	rs4149056	TT	32 (69.6)	1.15 (0.40–6.26)	0.689	10.72 (5.43–23.07)	0.635
		TC + CC	14 (30.4)	3.20 (0.19–4.13)		14.95 (9.36–19.97)	
	rs11045819	CC	39 (84.8)	2.18 (0.37–6.13)	0.850	12.88 (6.55–22.62)	0.999
		CA+ AA	7 (15.2)	0.89 (0.39–5.37)		10.32 (5.49–21.07)	
*SLCO1B3*	rs60140950	GG	39 (84.8)	2.18 (0.37–6.13)	0.850	12.88 (6.55–22.62)	0.999
		GC + CC	7 (15.2)	0.89 (0.39–5.37)		10.32 (5.49–21.07)	
*ABCB1*	rs1045642	AA	14 (30.4)	3.30 (0.47–6.40)	0.302	16.29 (9.87–23.86)	0.346
		AG + GG	32 (69.6)	1.44 (0.35–5.02)		11.29 (5.43–22.05)	
	rs9282564	TT	35 (76.1)	1.17 (0.35–5.47)	0.466	11.04 (5.49–22.62)	0.435
		CT + CC	11 (23.9)	3.01 (0.48–6.13)		15.98 (8.56–21.45)	
*NR1I2*	rs3814055	CC	19 (41.3)	2.18 (0.89–6.53)	0.746	15.98 (5.41–23.39)	0.917
		CT + TT	27 (58.7)	1.13 (0.19–5.47)		11.53 (6.55–22.25)	
	rs2276707	CC	29 (63.0)	3.01 (0.31–6.42)	0.354	15.98 (5.88–23.39)	0.510
		CT + TT	17 (37.0)	1.71 (0.43–3.33)		11.04 (6.15–18.04)	
	**rs3732357**	GG	7 (15.2)	3.16 (0.48–8.08)	0.077	22.25 (8.56–31.04)	**0.026**
		GA + AA	39 (84.8)	1.71 (0.35–5.37)		11.53 (5.41–21.07)	
	rs3732359	GG	6 (13.0)	3.02 (0.46–9.70)	0.211	19.12 (7.79–38.33)	0.058
		GA + AA	40 (87.0)	1.85 (0.35–5.44)		12.13 (5.70–21.36)	

^a^ SNP ID was extracted from the NCBI dbSNP database (https://www.ncbi.nlm.nih.gov/snp/; accessed on 8 May 2023). ^b^ The dominant genetic model was used to explore the association between the RIF pharmacokinetic parameters and selected SNPs. ^c^ Variables are presented as median (interquartile range). ^d^ Group comparison was performed using linear regression after controlling for biological sex, age, body weight, and rifampicin dose. A *p*-value of < 0.05 was considered statistically significant. Abbreviations: RIF—rifampicin; PK—pharmacokinetics; *SLCO1B1*—solute carrier organic anion transporter family member 1B1; *SLCO1B3*—solute carrier organic anion transporter family member 1B3; *ABCB1*—ATP binding cassette subfamily B member 1; *NR1I2*—nuclear receptor subfamily 1 group I member 2; SNP—single nucleotide polymorphism; C_max_—peak plasma concentration measured 2 h post-dose; AUC_0–6 h_—area under the time–concentration curve from 0 to 6 h post-dose.

**Table 4:** Comparison of genotype frequencies between patients in the DILI and non-DILI groups for selected SNPs.

Gene	SNP ID ^a^	Genotype ^b^	Genotype Frequency, n (%)		*p*-Value ^d^	OR (95% CI)
			DILI Group (n = 6) ^c^	Non-DILI Group (n = 40)		
*SLCO1B1*	rs2306283	AA	2 (33.3)	17 (42.5)	0.963	1.07 (0.05–22.80
		AG + GG	4 (66.6)	23 (57.5)		
	rs4149056	TT	3 (50.0)	29 (72.5)	0.571	2.49 (0.11–58.54)
		TC + CC	3 (50.0)	11 (27.5)		
	rs11045819	CC	5 (83.3)	34 (85.0)	0.230	8.27 (0.26–260.17)
		CA+ AA	1 (16.6)	6 (15.0)		
*SLCO1B3*	rs60140950	GG	5 (83.3)	34 (85.0)	0.230	8.27 (0.26–260.17)
		GC + CC	1 (16.6)	6 (15.0)		
*ABCB1*	rs1045642	AA	2 (33.3)	12 (30.0)	0.706	0.55 (0.02–12.68)
		AG + GG	4 (66.6)	28 (70.0)		
	rs9282564	TT	5 (83.3)	30 (75.0)	0.299	0.11 (0.002–7.00)
		CT + CC	1 (16.6)	10 (25.0)		
*NR1I2*	rs3814055	CC	3 (50.0)	16 (40.0)	0.447	0.28 (0.01–7.46)
		CT + TT	3 (50.0)	24 (60.0)		
	rs2276707	CC	4 (66.6)	25 (62.5)	0.681	0.55 (0.03–9.59)
		CT + TT	2 (33.3)	15 (37.5)		
	rs3732357	GG	0 (0.0)	7 (17.5)	0.997	N/A
		GA + AA	6 (100.0)	33 (82.5)		
	rs3732359	GG	0 (0.0)	6 (15.0)	0.998	N/A
		GA + AA	6 (100.0)	34 (85.0)		

^a^ SNP ID was extracted from the NCBI dbSNP database (https://www.ncbi.nlm.nih.gov/snp/; accessed on 8 May 2023). ^b^ The dominant genetic model was used to explore the association between anti-TB drug-induced liver injury and selected SNPs. ^c^ According to the CTLD clinical laboratory, DILI was defined as follows: ALAT > 60 U/L for males and > 45 U/L for females and ASAT > 55U/L for males and > 45 U/L for females and/or total bilirubin > 19.0 µmol/L and/or conjugated bilirubin > 3.4 µmol/L. ^d^ Group comparison was performed using logistic regression after controlling for biological sex, age, smoking and alcohol consumption status, and ALAT and ASAT level at the baseline. A *p*-value of < 0.05 was considered statistically significant. Abbreviations: *SLCO1B1*—solute carrier organic anion transporter family member 1B1; *SLCO1B3*—solute carrier organic anion transporter family member 1B3; *ABCB1*—ATP binding cassette subfamily B member 1; *NR1I2*—nuclear receptor subfamily 1 group I member 2; DILI—drug-induced liver injury; SNP—single nucleotide polymorphism; OR—Odds Ratio; 95% CI—95% Confidence Interval; N/A—not applicable; CTLD – Riga East University Hospital, Centre of Tuberculosis and Lung Diseases; ALAT—alanine aminotransferase; ASAT—aspartate aminotransferase.

## Data Availability

The datasets supporting the findings of this study are not publicly available due to ethical restrictions but are available from the corresponding author upon reasonable request.

## Supplementary Materials

The following supporting information can be downloaded at: https://www.mdpi.com/article/10.3390/pharmaceutics16030388/s1, Table S1: Sequences of the primers specifically designed for this study and used in the amplification step in the NGS workflow; Table S2: Characterization of the study population stratified by RIF plasma concentration 2 h post-dose, and comparison of patient characteristics between the patients with normal exposure (≥8 µg/mL) and underexposure (<8 µg/mL) 2 h post-dose; Table S3: Characterization of the study population stratified by DILI status and comparison of patient characteristics between DILI and non-DILI groups.
