# Supplementary material for: Exploring Variability in Rifampicin Plasma Exposure and Development of Anti-Tuberculosis Drug-Induced Liver Injury among Patients with Pulmonary Tuberculosis from the Pharmacogenetic Perspective
Source: Pharmaceutics. 2024 Mar 12;16(3):388. doi: 10.3390/pharmaceutics16030388 (PMC10974048; doi:10.3390/pharmaceutics16030388)
Supplement: Supplementary file 1 [file pharmaceutics-16-00388-s001.zip › Table S1_v2.pdf]

Table S1 Sequences of the primers specifically designed for this study and used in the amplification step in the NGS workflow

Primer name	Sequence (5'–3')	Genomic coordinates [a]	Product size, bp
AADAC_1F	AATCGCAGGGTGGAACACTT	chr3:151813557–151813576	5347
AADAC_1R	TAGGACTCAGGTGGGTTAGGT	chr3:151818883–151818903	
AADAC_4F	GTAATCACCTGCTGTTCGCT	chr3:151816407–151816426	4507
AADAC_4R	AAGCCTAGCACTCACTTGCAG	chr3:151820893–151820913	
AADAC_5F	CTGGGATGCGGGCAATGTTA	chr3:151824444–151824463	5101
AADAC_5R	TTACCTCAAATACAGCTCTTCTGC	chr3:151829521–151829544	
SLCO1B1_3F	TCAGAGCTGTGATAGCCGTTC	chr12:21129487–21129507	1886
SLCO1B1_2R	AGGGCTCAGAATGTAAGCGTG	chr12:21131350–21131370	
SLCO1B1_5F	GATGGTGGTAAGGTCAAGCAA	chr12:21140920–21140940	1437
SLCO1B1_5R	CTGAGTCTTGGCTTCTCATTTAGC	chr12:21142333–21142356	
SLCO1B1_6F	CCTCCCAGTTAGCCAATGCT	chr12:21172283–21172302	4102
SLCO1B1_6R	TTCACACACACGCTCTAATGTATG	chr12:21176361–21176384	
SLCO1B1_8F	TCTATTCCCGCAACCTCCAAG	chr12:21176049–21176069	1141
SLCO1B1_9R	GCAGTTGGCCCTGTTCAATC	chr12:21177170–21177189	
SLCO1B1_10F	GCTTGAGGTGATCTTCTGTGTGT	chr12:21195954–21195976	1504
SLCO1B1_10R	ACTGAACATTGCCATACGCC	chr12:21179486–21179505	
SLCO1B1_11F	TATGTTGAGCCAGGGAATAGAGC	chr12:21195954–21195976	5470
SLCO1B1_11R	AGGCTAGGTTACTGGGAGTCA	chr12:21201403–21201423	
SLCO1B1_12F	CCAACAAATATCCTCAGCCACAC	chr12:21201747–21201769	4761
SLCO1B1_12R	AAGGGTAGTGATCCCAGAGC	chr12:21206488-21206507	
SLCO1B1_13F	CCCTCTTTGCACACTAGCGA	chr12:21215995–21216014	1669
SLCO1B1_13R	TTTACCCTGAGAGATGCAAGGC	chr12:21217642–21217663	
SLCO1B1_14F	TTCAGAGGCATAATAAAGTCTGTTC	chr12:21221999–21222023	3395
SLCO1B1_14R	TTCCAGATGCCCATGCAGTT	chr12:21225374–21225393	
SLCO1B1_15F	GAAGGCCAGAGGCAACTAGA	chr12:21238757–21238776	2332
SLCO1B1_15R	AGAGACATACCAGTCTGATGATTGA	chr12:21241064–21241088	
SLCO1B3_1F	ACTCAACAGAGGGGTGTTTGG	chr12:20809310–20809330	1960
SLCO1B3_1R	CCCTCACCCTAAATCAAACGA	chr12:20811249–20811269	
SLCO1B3_4F	GGGCTGCTTATTACCTTGAGACCAC	chr12:20812980–20813004	4278
SLCO1B3_4R	TCTTACCCCAGAAACACAGGCAAC	chr12:20817234–20817257	
SLCO1B3_5F	TCTTTGCCTCCACAAAGTTCTAT	chr12:20854595–20854617	1505
SLCO1B3_5R	TGAGCCTGGGAGGTAAACAAG	chr12:20856079–20856099	
SLCO1B3_7F	TGATTCTATGGCTGCAACATGC	chr12:20857588–20857609	4341
SLCO1B3_7R	TGGATGTCATGGCTGTGATTATGA	chr12:20861905–20861928	
SLCO1B3_10F	CCTTAGAACTCACAGAGCGGT	chr12:20860454–20860474	4821
SLCO1B3_10R	AACAACTCAGGCAGACTTCAGG	chr12:20865253–20865274	
SLCO1B3_11F	CACATTCGCTCTGTGTTAAGCC	chr12:20874209–20874230	4452
SLCO1B3_11R	CCAGAAGCCTGTTCCAATGCT	chr12:20878640–20878660	
SLCO1B3_12F	AAGCATTGGAACAGGCTTCTG	chr12:20878639–20878659	5410
SLCO1B3_12R	GCTGTGGGTGAATTGAAAGCA	chr12:20884028–20884048	
SLCO1B3_13F	AGAGGTCTGCTAAGAATTACTCCTG	chr12:20898039–20898063	4662
SLCO1B3_13R	CCTTGCTGTTGAACTTTGCTTACA	chr12:20902677–20902700	
SLCO1B3_14F	AGACAGCTGGGGTTTAGTTTCTG	chr12:20915785–20915807	2529
SLCO1B3_14R	TACCATGGTGGTCTATCAAGGA	chr12:20918292–20918313	
ABCB1_1HF	TAGTCCTACAGCTTCACTACTGT	chr7:87501556–87501578	4374
ABCB1_1HR	GCCACGTCAGCTCTGGATAC	chr7:87505910–87505929	
ABCB1_2F	ACCTCAAATCCAGCTCACAGTA	chr7:87505386–87505407	4303
ABCB1_2R	GGTTGAAACACAGGAAACATGACAG	chr7:87509664–87509688	

ABCB1_3F	CTGTCATTGCTGACCACCATC	chr7:87514849–87514869	2231
ABCB1_3R	CTATTAGAGGCAAAGCTCTCTGGT	chr7:87517056–87517079	
ABCB1_4F	GAGGCAGAATCTTACCCACCTC	chr7:87518667–87518688	2973
ABCB1_4R	AACCCAAAGTCCCTTGAGCG	chr7:87521620–87521639	
ABCB1_5F	GCATGAGGTTTGTCCCCTACA	chr7:87529955–87529975	2085
ABCB1_5R	ATGCTTCAGACTCCCTCTGGAA	chr7:87532018–87532039	
ABCB1_6F	TTCACTCACTTTATTCCAGCCACT	chr7:87535301–87535324	2243
ABCB1_6R	AGCGACAGCTCCGTTCTACT	chr7:87537524–87537543	
ABCB1_7F	GGGAAGAAACACTCGTGCCC	chr7:87538903–87538922	2808
ABCB1_7R	TGACTCCAAGAGGGAATCCG	chr7:87541691–87541710	
ABCB1_8F	AGAACAGTGCATTCTCAAGCCA	chr7:87543745–87543766	3372
ABCB1_8R	GAGCATTGGGAAAGTTACAAAGGT	chr7:87547093–87547116	
ABCB1_9F	AGTGGCATTCAACACCTGGAA	chr7:87548914–87548934	1748
ABCB1_9R	GTCACTTTATCCAGCTCTCCACA	chr7:87550639–87550661	
ABCB1_10F	TCTAGCTCGCATGGGTCATC	chr7:87550147–87550166	1767
ABCB1_10R	AACTGGGTCTTCTGACTCCTCT	chr7:87551892–87551913	
ABCB1_11F	AAAGTTTAGCTGGCTGCCCTA	chr7:87552422–87552442	2853
ABCB1_11R	GCAGGGAGTTGCGATTAAGA	chr7:87555255–87555274	
ABCB1_12F	CCTTTGAACTATGGCACTTAACCAT	chr7:87560269–87560293	2561
ABCB1_12R	TCACACCTGGCCTTTTCATATCC	chr7:87562807–87562829	
ABCB1_13F	ACATACTGGGCCAATTTCTCTACTG	chr7:87565314–87565338	2378
ABCB1_13R	GAAGGGCCACTATTTGATTCGTT	chr7:87567669–87567691	
ABCB1_14F	ACCTACACGTACCTTTGGACA	chr7:87569418–87569438	1500
ABCB1_14R	GCCACTAAATGTAAACTATGACTCC	chr7:87570893–87570917	
ABCB1_15F	AGCAGTAAGAGAGCTAACGCC	chr7:87583920–87583940	2867
ABCB1_15R	CACTCGGCAGTCCAGTACC	chr7:87586768–87586786	
ABCB1_16F	CACCAAAATTCCAAAGGGCG	chr7:87594679–87594698	2177
ABCB1_16R	TGCCTTTACTGGGAATTGGGT	chr7:87596835–87596855	
ABCB1_17F	GCCATTTACCGGAAGCAAGA	chr7:87599424–87599443	4211
ABCB1_17R	CCCAGTGAGTTTGGCTGAAATG	chr7:87603613–87603634	
ABCB1_18BF	AGGTTCTATTGGTCGGAGCTG	chr7:87711806–87711826	3372
ABCB1_18BR	CCCTCATCCTTTATAGCATTTCAC	chr7:87715154–87715177	
NR1I2_1F	TGAACTGAGCAGCCACCAAAT	chr3:119778867–119778887	4982
NR1I2_1R	AGAACAACATGGCAACAGATAGG	chr3:119783826–119783848	
NR1I2_2F	TCCATGATCCGACCCAGACA	chr3:119806918–119806937	3836
NR1I2_2R	ACACCTAGCCTTTTCCGCAT	chr3:119810734–119810753	
NR1I2_3F	ATGCGGAAAAGGCTAGGTGTT	chr3:119810734–119810754	2819
NR1I2_3R	ACCTGGCACCATCCCTAATC	chr3:119813533–199813533	
NR1I2_4F	CAAGCTCAGTGGGTGGAGTT	chr3:119814732–119814751	1421
NR1I2_4R	TGGCCTTAACCCTTCATGGC	chr3:119816133–199816152	
NR1I2_5F	CCCTAACCTCCACAACGACTC	chr3:119816398–119816418	3263
NR1I2_5R	CCTATGTGAACTCTTCAGGGCAT	chr3:119819638–119819660	

[a] Reference genome: GRCh38.p13, GCF 000001405.39.
