# Supplementary material for: Exploring Variability in Rifampicin Plasma Exposure and Development of Anti-Tuberculosis Drug-Induced Liver Injury among Patients with Pulmonary Tuberculosis from the Pharmacogenetic Perspective
Source: Pharmaceutics. 2024 Mar 12;16(3):388. doi: 10.3390/pharmaceutics16030388 (PMC10974048; doi:10.3390/pharmaceutics16030388)
Supplement: Supplementary file 1 [file pharmaceutics-16-00388-s001.zip › Table S2_v2.pdf]

Table S2 Characterisation of the study population stratified by RIF plasma concentration 2 hours post-dose, and comparison of patient characteristics between the patients with normal exposure (≥ 8 µg/mL) and underexposure (< 8 µg/mL) 2 hours post-dose

Characteristic		RIF exposure [a]				
		Normal exposure (n = 4)		Underexposure (n = 42)		*p*-value
		No./Total (%)	Median (IQR)	No./Total (%)	Median (IQR)	
Biological sex	Male	4/4 (100.0)		31/42 (73.8)		0.559 [c]
	Female	0/4 (0.0)		11/42 (26.2)		
Age, years	Overall		45 (36–52)		47 (38–56)	0.721 [d]
	< 60 years	4/4 (100.0)		37/42 (88.1)		1.000 [c]
	≥ 60 years	0/4 (0.0)		5/42 (11.9)		
Body weight, kg	Overall		54 (51–60)		65 (57–74)	**0.021** [d]
BMI [b]	Underweight	2/4 (50.0)		9/42 (21.4)		0.470 [c]
	Normal weight	2/4 (50.0)		25/42 (59.5)		
	Overweight	0 (0.0)		8/42 (19.0)		
Smoking status	Smoker	4/4 (100.0)			31/42 (73.8)	0.559 [c]
	Non-smoker	0/4 (0.0)			11/42 (26.2)	
Increased alcohol consumption	Yes	1/4 (25.0)		14/42 (33.3)		1.000 [c]
	No	3/4 (75.0)		28/42 (66.7)		
RIF dose, mg/kg	Overall		11.2 (10.1–11.7)		9.2 (8.1–10.6)	**0.021** [d]

[a] Based on the RIF C_{max} values at 2h, according to the reference range suggested by Alsultan et al. [15]. Normal exposure: ≥ 8 µg/mL; underexposure: < 8 µg/mL.
[b] According to the World Health Organization recommendations [56], a patient was classified as underweight if the BMI was < 18.5 kg/m^2 and overweight if the BMI was ≥ 25.0 kg/m^2.
[c] Group comparison was performed using the Fisher's exact test.
[d] Group comparison was performed using the Mann-Whitney U test.
For all tests, a *p*-value of < 0.05 was considered statistically significant.
Abbreviations: RIF – rifampicin; BMI – body mass index.
