# Supplementary material for: Exploring Variability in Rifampicin Plasma Exposure and Development of Anti-Tuberculosis Drug-Induced Liver Injury among Patients with Pulmonary Tuberculosis from the Pharmacogenetic Perspective
Source: Pharmaceutics. 2024 Mar 12;16(3):388. doi: 10.3390/pharmaceutics16030388 (PMC10974048; doi:10.3390/pharmaceutics16030388)
Supplement: Supplementary file 1 [file pharmaceutics-16-00388-s001.zip › Table S3_v3.pdf]

Table S3 Characterisation of the study population stratified by DILI status and comparison of patient characteristics between DILI and non-DILI groups

		DILI group (n = 6) [b]		Non-DILI group (n = 40)		*p*-value
		No./Total (%)	Median (IQR)	No./Total (%)	Median (IQR)	
Demographic and anthropometric characteristics						
Biological sex	Male	3/6 (50.0)		32/40 (80.0)		0.138 [e]
	Female	3/6 (50.0)		8/40 (20.0)		
Age, years	Overall		50 (44–58)		46 (35–55)	0.345 [f]
	< 60 years	5/6 (83.3)		36/40 (90.0)		0.520 [e]
	≥ 60 years	1/6 (16.7)		4/40 (10.0)		
Body weight, kg	Overall		65 (52–69)		64 (55–74)	0.453 [f]
BMI [a]	Underweight	1 (16.7)		10 (25.0)		1.000 [e]
	Normal weight	4 (66.7)		23 (57.5)		
	Overweight	1 (16.7)		7 (17.5)		
Self-reported lifestyle factors						
Smoking status	Smoker	4 (66.7)		31 (77.5)		0.619 [e]
	Non-smoker	2 (33.3)		9 (22.5)		
Increased alcohol consumption	Yes	3 (50.0)		12 (30.0)		0.375 [e]
	No	3 (50.0)		28 (70.0)		
Baseline blood biochemical parameters						
ALAT, U/L			22 (13–52)		17 (12–23)	0.329 [f]
ASAT, U/L			18 (15–80) [c]		18 (15–27) [d]	0.914 [f]
Total bilirubin, µmol/L			7.4 (5.0–12.5) [c]		6.3 (5.2–8.4)	0.661 [f]
Conjugated bilirubin, µmol/L			3.4 (2.2–5.9) [c]		3.1 (2.4–4.0) [d]	1.000 [f]
Blood biochemical parameters after 10–12 days of anti-TB treatment						
ALAT, U/L			247 (157–972)		16 (13–21)	**< 0.001** [f]
ASAT, U/L			230 (125–722)		21 (17–26)	**< 0.001** [f]
Total bilirubin, µmol/L			6.1 (4.0–15.8)		4.6 (3.1–6.0)	0.108 [f]
Conjugated bilirubin, µmol/L			3.4 (3.1–13.6)		2.5 (1.7–3.5)	0.055 [f]
Rifampicin-related data						
RIF dose, mg/kg			9.3 (8.7–11.6)		9.4 (8.1–10.9)	0.453 [f]
C_{max}, µg/mL			2.15 (0.31–4.55)		2.09 (0.40–5.96)	0.987 [f]
$AUC_{0–6h}$, µg × h/mL			14.22 (9.43–22.81)		12.75 (5.43–22.53)	0.622 [f]

[a] According to WHO recommendations [56], a patient was classified as underweight if the BMI was < 18.5 kg/m^2 and overweight if the BMI was ≥ 25.0 kg/m^2.
[b] According to the CTLD clinical laboratory, DILI was defined as follows: ALAT > 60 U/L for males and > 45 U/L for females and ASAT > 55U/L for males and > 45 U/L for females and/or total bilirubin > 19.0 µmol/L and/or conjugated bilirubin > 3.4 µmol/L.
[c] Data were available for 5 patients.
[d] Data were available for 39 patients.
[e] Group comparison was performed using the Fisher's exact test.
[f] Group comparison was performed using the Mann-Whitney U test.
For all tests, a *p*-value of < 0.05 was considered statistically significant.
Abbreviations: WHO – World Health Organization; BMI – body mass index; CTLD – Riga East University Hospital, Centre of Tuberculosis and Lung Diseases; DILI – drug-induced liver injury; ALAT – alanine aminotransferase; ASAT – aspartate aminotransferase; RIF – rifampicin; C_{max} – peak plasma concentration measured 2 hours post-dose; $AUC_{0–6h}$ – area under the time-concentration curve from 0 to 6 hours post-dose.
